# Formal and informal science advice in emergencies: COVID-19 in the UK

**DOI:** 10.1098/rsfs.2021.0059

**Published:** 2021-10-12

**Authors:** Christopher J. M. Whitty, Luke B. Collet-Fenson

**Affiliations:** Department of Health and Social Care, 39 Victoria Street, London, UK

**Keywords:** COVID-19, scientific policy, emergencies

## Abstract

The importance of scientific advice to government gains greater recognition in emergencies but inevitably has to be done in an environment of uncertainty, with limited data and at high speed. Adapting existing structures is more effective than creating new ones in an emergency. Between emergencies, the UK has a structured scientific advice system, including Chief Scientific Advisers, scientists in government, regulatory bodies and independent expert committees, which were adapted to COVID-19 under the umbrella of the Scientific Advisory Group for Emergencies. These worked alongside networks of informal scientific advice, including internationally. Multiple sciences were needed, including from the social sciences and engineering in addition to clinical science and epidemiology, and these had to be integrated. A centrally directed clinical research programme helped provide practitioners robust evidence, with observational and interventional trials providing data for policy and testing treatments and vaccines. The scale of the emergency meant unavoidable tension between detailed work and speed, and between an integrated scientific view usable in decision-making and constructive challenge. While a final judgement of the UK scientific response will take time, everyone should be grateful to the thousands of scientists involved for the research, synthesis and advice, which improved outcomes for the public.

## 1. Introduction

Governments and other agencies need science in emergencies. This may seem an obvious point to the readers of this journal but the use of science in many areas of policy between emergencies is patchy and its importance is not always appreciated in all areas of government. In the great majority of natural emergencies including epidemics and pandemics; geological emergencies including earthquakes and volcanoes; metrological emergencies such as floods, droughts and major storms, an effective response is, however, widely recognized to require a strong scientific foundation. Good science does not in any way guarantee a good outcome, and in particular rapid policy and excellent operational delivery is essential, but without a strong scientific foundation an ineffective response is highly likely.

Emergencies by definition require science to be undertaken at a pace much faster than is usual in academic work, using absent, imperfect or gradually emerging data. They also always need a range of scientific disciplines much wider than is popularly understood, or indeed predicted before the emergency occurs [1]. Almost all major emergencies depend as much on human behaviour as biological or physical factors and the social sciences including behavioural science, anthropology and economics should have a central part in the response. In the current crisis in the UK, sciences from the public sector that have been central to the response have included virology, vaccine and immunological science, clinical science including trials, epidemiology and mathematical modelling, engineering, and behavioural psychology. Science from the private sector including pharmaceutical and devices industries have also been essential, and diagnostics, drugs and vaccines would not have been available without them.

Much of the science that has been needed for COVID-19 has not had government as its principal end user. Clinical practitioners, manufacturers and the general public have often been the main users of the science that has come out. Government is still often central to part of this response, whether as a funder, in prioritization, regulation or in coordination, but much of this is science organized in the normal way through conventional academic or life sciences pathways, albeit at a much faster speed than is usual.

COVID-19 is the largest global emergency for a generation. It has tested the link between scientific expertise and data, policy and practice. In this paper, we will describe both the structure by which the UK government got its scientific advice, and the interaction between science and government in the wider scientific and research effort. It is too early to say confidently which areas of the system worked well and which did not, this will become clearer as the pandemic moves to a more predictable phase. Early judgements are often revised as a fuller picture emerges. This paper is heavy on structures for a scientific journal, because the complexity and scale of the scientific architecture, and how it interacts is often not fully understood outside government (or indeed within it). It is a repeated finding in emergencies that adapting functioning existing structures, where available, is usually more effective that setting them up from scratch, especially in the early most uncertain and most fast-paced response.

## 2. Initial science aimed at government

The UK has a relatively structured scientific advice system for the ordinary run of business between emergencies, and additional separate scientific structures designed to respond to emergencies, although not used on this scale for some decades. Almost every government department has a Chief Scientific Adviser, usually a senior (National Academy-level) scientist seconded into government from academia. Many are supported by departmental scientific advisory boards of senior academics. There is a central Government Office for Science led by a senior scientist who is the Government Chief Scientific Adviser (GCSA); they coordinate the work of the Chief Scientific Advisers across government [2].

The UK government, in common with most other governments, has a network of scientific laboratories and other employed scientists and they played a major role in the response, such as the development of initial diagnostic tests at Public Health England laboratories at Porton Down and initial epidemiological studies [3]. In addition, the regulatory scientific bodies, such as the Medicines and Healthcare products Regulatory Agency (MHRA), were central to the assessment and licencing of drugs, vaccines and diagnostic tests for post-trial use.

Supporting this group of scientists who are employed within government or its agencies is a network of scientific advisory committees covering different areas of science relevant to government. Most are independent of government and chaired by an independent senior scientist from academia, but report through to it. Some are internal to government but use external expertise. Examples of these that were relevant to the initial COVID-19 response were the New and Emerging Respiratory Virus Threats Advisory Group (NERVTAG) which advises on the science of new respiratory viruses, the Advisory Committee on Dangerous Pathogens (ACDP) which looks at laboratory and clinical safety for new infections, the Scientific Pandemic Influenza Group on Modelling (SPI-M), the Independent Scientific Pandemic Insights Group on Behaviours (SPI-B) and the Joint Committee on Vaccination and Immunisation (JCVI). All of these pre-existing committees have scientists who are domain experts in relevant fields, mainly independent academics, with a scientific secretariat within government.

An example is NERVTAG, one of the first expert committees to consider COVID-19, meeting first on the 13 January 2020. Early in the pandemic, with limited data, NERVTAG, which included clinicians, virologists and epidemiologists, provided initial advice on clinical assumptions including on infection attack rate, duration of hospitalization and case fatality rate [4]. The data were used by clinicians and public health practitioners, but SPI-M also used NERVTAG clinical assumptions to underpin their modelling. This joint working between expert committees with different expertise has been essential to the COVID-19 response.

SPI-M's consensus views brought together the modelling outputs [5,6] and shaped the initial response. Given the sensitivity of modelling to assumptions made, and the wide panel of possible models, it is vital to have SPI-M, who bring together the different modelling groups and present a consensus view rather than rely on a single model. This became increasingly sophisticated as the pandemic progressed to inform later stages of planning [7].

Feeding into these formal committees was a very large network of scientists who undertook rapid response research, analysis, modelling and commentary to inform the committee structure. Most of this work was unpaid and done well outside normal working hours and the work of the scientists involved has been remarkable.

While these groups exist between emergencies, the main structure for emergencies that need scientific input of any sort in the UK is the Scientific Advisory Group for Emergencies (SAGE). This has no standing membership and is set up with relevant experts from within and outside government for any emergency that requires significant scientific advice on a cross-government basis. It exists to ensure government can integrate science from multiple groups, and that a single version of the science, presented with appropriate levels of confidence and outlier opinion if relevant, is presented to policymakers rather than each department working to a different model. It is chaired by the GCSA, and in health emergencies co-chaired by the Chief Medical Officer (CMO). The participants in SAGE are chosen for the specific emergency SAGE has been constituted to provide advice on, and in this emergency, the membership has evolved as the science problems government needs answers to change. The Government Office for Science and individual departments have lists of experts who can be called on in an emergency who tend to be the earlier members but later members were chosen for specific skill gaps. SAGE took advice both from the standing committees and *ad hoc* committees that considered issues such as care homes or schools. While SAGE main committee members generally have to have some generalist science skills to incorporate science from many disciplines in addition to their own specialism, specialist sub-committees have deep subject experts in particular fields.

One *ad hoc* committee created due to need by SAGE is the Environmental Modelling Group (EMG), which looks at transmission routes mechanistically, and potential countermeasures, and included engineering as well as conventional medical expertise. EMG provided advice to the Department for Business Energy and Industrial Strategy to inform their guidance to workplaces. EMG papers in particular emphasized the importance of ventilation and how to achieve it [8].

Alongside this complex formal advisory structure was inevitably an informal network of advice for virtually every senior scientist involved in the response, including senior government scientists. There was also a wide structure of formal and informal links between international scientists who were advising their respective governments. Formal groups included regular meetings convened by the World Health Organization of senior public health scientists from all continents, and a regular informal meeting of European chief scientific advisers and equivalents convened by the UK GCSA. Alongside these were bilateral interactions which could be regular (for example between the UK and USA) or followed the course of the pandemic.

In the initial phases of the global pandemic where the majority of infection was in East Asia, scientists from around the world, including the UK, learned from scientists in China, South Korea, Singapore and Japan among others. When the UK had the first major outbreak of Alpha variant scientists from other countries contacted UK government scientists to get an early fix on this new threat. In turn, scientists from India kindly provided very important information on the Delta variant in advance of publications. The extent of international interaction between government scientists is probably underappreciated by the wider community. The generosity of time of those scientists who were simultaneously leading their own national response at the leading edge of the pandemic and advising their international peers who were further behind any given epidemic curve was remarkable. This started with the clinical and scientific advice given bilaterally and multilaterally by scientists from China in the initial few weeks which was essential to the international and UK response.

While informal advisory networks have a major role, publications are the best form of communication in science as they are available to all, present methodology and data in a structured way and can receive rapid peer review whether formal or informal. For policy as well as research, the widespread use of preprints, previously relatively rare in medical research, was an important intermediate step between informal reporting of emerging data and a fully peer reviewed article. In a very rapidly developing field where we were often starting from minimal knowledge having the 80% finished article available rapidly was potentially policy changing and life-saving. For all its well-recognized faults, peer review is an incredibly powerful tool for ensuring high-quality science is presented and less high-quality science improved or not published. It can however add significant delay, and in a very rapidly moving emergency delay can be fatal. The rapid uploading of data to sites such as GISAID, which hosts much of the genomic data, has been absolutely essential. As an example, the RECOVERY trial team announced dexamethasone reduced mortality in a statement on 16 June 2020 [9], a preprint was published on 17 July 2020 and the paper was published with peer-review on 25 February 2021 [10]. Government advice and clinical practice changed well before the paper.

Much of the science informing government was neither commissioned nor coordinated by government, but by conventional investigator-led scientific publication. Informal groupings have often been very useful in informing or challenging government advice; a good example is the COVID-19 Actuaries Response Group [11]. There was a constant stream of commentary from the scientific community (in the broadest sense), some critical. This was often very useful in challenging assumptions and tended to have greater influence when backed up by data and analysis.

## 3. Initial science aimed at practitioners

Government makes society level policy decisions but decisions at an individual level are made by practitioners. For example government agencies such as MHRA licence drugs and vaccines based on scientific advice, but it is an individual practitioner who prescribes them and decides who will benefit. For clinical medicine, the biggest driver of practice is clinical trials. There was a very strong risk that so many trials started that none of them had sufficient statistical power to reach convincing points.

The UK has a very centralized health delivery system through the National Health Service (NHS), and three major funders of clinical research: the National Institute for Health Research (NIHR), the Medical Research Council (MRC) part of UK Research and Innovation (UKRI), and the Wellcome Trust. It was therefore well situated for the government funders of research NIHR and MRC, to coordinate which research was prioritized, and use the NHS and existing NIHR networks to deliver this. The ‘Urgent Public Health’ badging system aimed support, such as research nurses, at the priority research [12] and the UK CMOs discouraged the use of off-licence treatments outside of a trial where participation in a trial was possible [13]. This helped trials achieve endpoints and provide practitioners with robust evidence.

The possibility that such research would be needed in a pandemic or major epidemic had been anticipated for some time. Observational studies such as the COVID-19 Clinical Information Network (CO-CIN) and SARS-CoV-2 Immunity and Reinfection Evaluation (SIREN) studies, which looked at disease in hospitalized cases and infection in healthcare workers, respectively, were relatively rapidly stood up. CO-CIN provided early data on important issues, such as comorbidity and ethnicity [14,15]. SIREN provided data on natural immunity [16] and vaccination [17].

The RECOVERY clinical trials platform which was activated in advance of the first wave in the UK proved highly successful demonstrating existing drugs which worked to reduce mortality such as dexamethasone [10], or did not work such as hydroxychloroquine [18]. To select the most promising drugs, RECOVERY took its initial list from NERVTAG and then a specialist group, the UK COVID-19 Therapeutics Advisory Panel was set up to provide further science-led recommendations [19].

An existing programme of work to develop a MERS coronavirus vaccine in the University of Oxford, which had been prioritized for funding by the UK Vaccines Network swung over to address COVID-19 and became the very successful AZ vaccine, primed by early COVID-19 government funding [20].

A genomics consortium was central to the assessment of importation of cases, and identifying and tracking variants (COVID-19 Genomics UK Consortium, https://www.cogconsortium.uk/).

## 4. Tension between aims in the science advice system in a major emergency

COVID-19 is the largest and longest lasting emergency for at least a generation, affecting the lives of every citizen and taking the lives of over 120 000 so far in the UK alone. In emergencies, adapting existing structures is almost always more effective than having to set them up from scratch. This paper has described some (but by no means all) of the scientific structures which fed scientific advice into one government (UK) during the COVID-19 pandemic to date. Supporting that were thousands of scientists in hundreds of research groups from multiple disciplines. Within the UK individual nations made slightly different policy decisions based on that advice, and some (e.g. Scotland) developed their own scientific structures which took the outputs of the four-nation UK advice and adapted it for local epidemiological and operational conditions.

Several tensions exist in trying to provide science advice in an emergency on this scale. There is an obvious tension between comprehensive advice that has been rigorously tested against speed. The time between the first international reports of a completely new infection, COVID-19 and the first UK wave was less than 12 weeks. The short time between novel infection emerging and action being required was a problem shared globally. There is also an important tension between providing non-scientific policymakers and the public multiple versions of the science and asking them to choose, which leads to chaos, and a single agreed (ideally consensus) version with appropriate cautions, which is much easier for policymakers to use but run the well-known dangers of groupthink or a monopoly of ideas. Widespread media use of science voices (broadly defined) both informed the public, which was essential, and caused confusion. The playing out of scientific debates in the media, often using scientific voices which were representative of relatively fringe (although usually perfectly respectable) opposing views sometimes provided a healthy challenge but did not always advance public or policymaker understanding. It is positive that the public probably now have a greater awareness that science advice is based on the currently available data and will change if that data change, but explaining uncertainty will continue to be an important feature in ensuring trust in COVID-19 science advice, and science advice more broadly.

A robust assessment of the UK scientific response overall will take time and in our view will change substantially over that time; many of the initial judgements are partial. The work of the individual scientists who contributed to it through original research, scientific synthesis or contributing to specialist committees was however extraordinary in its speed, quality and commitment to improving outcomes for the public. Everyone should be grateful to the thousands of scientists involved.

## Data accessibility

This article has no additional data.
